# Case of Retained Menstrual Matter from Occluded Os

**Published:** 1879-05

**Authors:** E. Griffin

**Affiliations:** Atlanta, Ga.


ATLANTA
Medical and Surgical Journal.

VOL. XVII.] MAY—1879. [No. 2

### Original Communications.

### A CASE OF RETAINED MENSTRUAL MATTER FROM OCCLUDED OS.

By E. GRIFFIN, M.D., Atlanta, Ga.

I was called to see the following case, two months ago, when I gathered a history of the disease of the uterus that had existed for a year previously. No clear statement as to the character of the trouble, however, could be elicited; and the difficulties to be related were attributed to a combined attack of corporal and cervical metritis.

Some pain had always been present in menstruation, from the commencement of the attack, but the periods were otherwise regular until about five months ago. At this time she failed altogether to have the discharge, but feeling not unusually bad, supposed that everything would come all right, and felt comparatively easy. At the next period, however, no "sign" having shown itself, she became somewhat alarmed, and imagined more difficulty than really existed. About two months after she came under my charge, the following facts were obtained: Married white lady, multipara, aged thirty. About four months previously, ceased to menstruate, and at each recurring period, considerable pain was felt in the umbilical region. Digital examination revealed an ovoid, globular uterus with complete extinction of the cervix, apparently, and occlusion of the os, scarcely any vestige remaining. Palpation further revealed the fact that the walls were of high tension, a condition readily to be expected from the complete obliteration of the cervix that was patent upon digital touch. Examination per speculum confirmed the digital touch, and the globular form with the distended condition was marked. The theory of retained menstrual flow was at once concluded, and the uterine canal, obeying the ordinary law, underwent "retrograde dilatation behind the seat of stricture," resulting in the marked distension. It was also concluded that regurgitation might possibly be imminent, and for this reason operative interference was deemed advisable at once.

It has been the usual practice, I believe, to use in these cases a suitable trochar, but on the one in question, not having the trochar at hand, I used a bistoury. With this I made a crucial incision in the direction of the cervical canal, which was followed by the discharge of the contained matter. The character of this was of a dark, grumous, and very offensive fluid, and amounted in quantity to about two pints. Upon its discharge the patient expressed great relief, while the parts assumed, in a manner, their natural form. The discharge, in small amount, kept up some three or four days, and when the patient was last seen the cure seemed to have been complete.

It is not altogether an unusual occurrence to meet obstruction to the free flow of the menstrual discharge, I believe, but there is ordinarily some concatenation of symptoms that lead a moderately close observer to suspect the real condition. Tendencies to the malady exist from constantly maintained irritation or inflammation, but the stenosis is of comparatively slow progress—the entire occlusion being gradually accomplished. In this case, however, it seemed, from the history, that menstruation was perfect up to the period that it was entirely retained.

Cases have been reported where this condition had existed for months, so nearly similating pregnancy that the victim was condemned not only to suffer the discomfort of the disease, but to be subjected to the suspicion of moral irregularity.

Again, we may have the serious complication of regurgitation of the retained fluid in the peritoneal cavity. In such instances, the irritable uterus resenting the distension arising from the mass, spasmodically contracts, and drives it either through its own walls, producing rupture, or else by way of the cornua on through the fallopian tubes. "Ruysch, Haller and Brodie all believed that blood could flow back from the uterus into the peritoneum."

The manner of operating was beneficial, while, to myself, it was novel. Had there been a trochar at hand I should have used it; but, as events proved, it could have served no better end than the means adopted.

There are many, no doubt, to whom this instance may be commonplace, and yet there may be a few to whom the relation may be serviceable and interesting. Should this report have any avail, either in doing good or inducing reports of personal experience from other members of the profession, I shall feel fully compensated.

